# Autochthonous dairy goat breeds showed better milk quality than Saanen under the same environmental conditions

**DOI:** 10.5194/aab-62-83-2019

**Published:** 2019-02-28

**Authors:** Sarah Currò, Carmen L. Manuelian, Massimo De Marchi, Pasquale De Palo, Salvatore Claps, Aristide Maggiolino, Giuseppe Campanile, Domenico Rufrano, Annunziata Fontana, Giuseppina Pedota, Gianluca Neglia

**Affiliations:** 1 Department of Agronomy, Food, Natural Resources, Animals and Environment (DAFNAE), University of Padova, Legnaro (PD), 35020, Italy; 2 Department of Veterinary Medicine, University of Bari Aldo Moro, Valenzano (BA), 70010, Italy; 3 Council for Agricultural Research and Economics, Research Centre for Animal Production and Acquaculture (CREA-ZA), Muro Lucano (PZ), 85054, Italy; 4 Department of Veterinary Medicine and Animal Production (DMVPA), University of Naples Federico II, Naples, 80137, Italy; 5 Associazione Nazionale Allevatori, Rome, 00161, Italy

## Abstract

Studies on goat milk have mainly focused on cosmopolitan breeds and very limited information is available on local breeds, which is important for biodiversity preservation and local cheese production. The aim of this study was to evaluate the breed effect on milk yield, composition and somatic cell score (SCS) of five local Italian goat breeds (Garganica, Girgentana, Jonica, Maltese and Mediterranean Red) compared with a cosmopolitan specialized dairy breed (Saanen). A total of 60 goats (10 per breed) from an experimental farm were enrolled in the study. Milk yield, composition and SCS were recorded and analyzed every 2 weeks during the entire lactation. Data were analyzed using a mixed model with repeated measures. Saanen yielded between 0.27 and 0.62 kg day${}^{-1}$ more milk than the local breeds. Among local breeds, Maltese and Jonica were the most productive, with an average of 1.28 and 1.25 kg day${}^{-1}$, respectively, while Mediterranean Red, Garganica and Girgentana produced ≤1 kg day${}^{-1}$. Saanen had the highest SCS (6.81) and the lowest fat content (3.26 %). In relation to protein, Garganica showed the greatest content (3.71 %), and Saanen had a similar content to other local breeds (3.42 %) except for Maltese, which was lower (3.11 %). Saanen and Garganica had the lowest lactose percentage (4.28 % and 4.26 %, respectively). All breeds followed a similar pattern across lactation: SCS and fat and protein content peaked at the end of the lactation, whereas lactose percentage was highest at the beginning of the lactation. Differences between Saanen and the local breeds for milk yield, composition and SCS were consistent across lactation. In conclusion, local breeds produced less milk but with lower SCS and greater fat and lactose content than the Saanen cosmopolitan breed, suggesting a better milk quality.

## 1 Introduction

In the last 10 years, goat (*Capra hircus*) milk world production has increased by 27.9 %, from $14\times 10^{6}$ t in 2004 to more than $18\times 10^{6}$ t in 2014, and goat cheese manufacture has increased by 17 %, from $446\times 10^{3}$ t in 2004 to $523\times 10^{3}$ t in 2014 (FAOSTAT, 2019). The goat milk casein profile is more similar to human milk (Haenlein, 2004; Albenzio et al., 2012) than cow's milk is. Moreover, goat milk has greater protein micelles and smaller fat globules and a more favorable fat composition (Park, 1994; Williams, 2000; Faye and Konuspayeva, 2012), which is beneficial for digestibility and energy uptake (Park, 1994; Williams, 2000) as well as for cheese manufacture (Faye and Konuspayeva, 2012). However, the goat milk industry has put great efforts into increasing milk production, which has drastically eroded genetic variability in this species because farmers have often replaced local populations with genetically improved breeds to increase farm profitability.

Studies on milk yield and composition have mainly focused on cosmopolitan dairy-specialized goat breeds (Gipson and Grossman, 1989; Sung et al., 1999; Goetsch et al., 2011), and only few studies have investigated Italian local breeds (Tripaldi et al., 1998; Sacchi et al., 2005; Carnicella et al., 2008). The lack of information on potential milk yield and composition of autochthonous breeds is probably a major reason for the substitution of local with more productive cosmopolitan breeds such as Alpine, Murciano-Granadina and Saanen (Benjelloun et al., 2015). Currently, and according to official national data reported by the FAO (DAD-IS, 2019), Italy has 55 goat breeds considered of regional or local distribution, 34 of them being at risk of extinction and 16 having an unknown risk. The five most important breeds reared in south Italy are the Garganica, Girgentana, Jonica, Maltese and Mediterranean Red breeds, which are all at risk of extinction except for the Garganica, for which the risk level is unknown. Therefore, the aim of the present study was to evaluate the breed effect on milk yield, composition and somatic cell score (SCS) of five local goat breeds (Garganica, Girgentana, Jonica, Maltese and Mediterranean Red) compared with a cosmopolitan dairy specialized goat breed (Saanen) reared on the same experimental farm.

## 2 Material and methods

### 2.1 Animals and management condition

The study was carried out at the experimental farm of the Council for Agricultural Research and Economics (CREA, Potenza, Italy) from February to September 2016 and included Garganica (GA), Girgentana (GI), Jonica (JO), Maltese (MA), Mediterranean Red (MR) and Saanen (SA) goat breeds reared under the same management and feeding conditions. To our knowledge, there are not previous studies that included six different breeds reared under the same conditions allowing a direct comparison of their performance. A general description of the six breeds is reported in Table 1. Experimental procedures and animal care conditions followed the recommendations of European Union directive 86/609/EEC (CEU, 1986). A total of 60 dairy goats (10 does per breed) with a body condition score between 2.5 and 3.0 (1, very thin, to 5, very fat, with a 0.5 point increment; Villaquiran et al., 2005) and a body weight of 48±4 kg for GA, 42±6 kg for GI, 47±6 kg for JO, 46±5 kg for MA, 48±3 kg for MR and 64±7 kg for SA breeds at the beginning of the study were enrolled in the study. All goats and breeds grazed together during the day (8 h day${}^{-1}$) in a natural pasture and received hay (composition: 60 %–65 % of grasses and 40 %–35 % of legumes and others; chemical composition: 89.10 % of dry matter, 15.10 % of crude protein, 52.60 % of neutral detergent fibre and 1.10 Mcal kg${}^{-1}$ of net energy of lactation) ad libitum as a complement in the shelter. Moreover, goats were supplemented with commercial concentrate (chemical composition: 88.20 % of dry matter, 21.70 % of crude protein, 23.00 % of neutral detergent fibre and 1.77 Mcal kg${}^{-1}$ of net energy of lactation) in the milking parlour according to their requirements considering the mean body weight and mean milk production for each breed following NRC (2007) recommendations; this was adjusted every 15 days throughout lactation. All selected does kidded twins in February and parities from 1 to 5 were balanced between breeds (i.e., the same number of goats for each parity for each breed). Kids were kept with their dams until 40 days from birth and temporally separated from their dams 24 h before every sampling day. All goats were mechanically milked twice a day (07:30 and 17:30 LT) in a double 24-stall herringbone low-line milk pipeline milking parlour (Alfa Laval Agri; Monza, Italy) equipped with recording jars and electronic pulsators at a vacuum of 38 kPa, 90 pulses min${}^{-1}$ and a 60 % pulsation ratio. Pre-milking included only forestripping, without any preparation of udder and teats. None of the does presented mastitis throughout the study.

**Table 1:** Origin and description of goat breeds included in the study retrieved from Capre.it (2019) unless otherwise indicated.

Trait	Garganica	Girgentana	Jonica	Maltese	Mediterranean Red	Saanen
Origin	Italy	Italy	Italy	Italy	Italy	Switzerland
Herd	Big and medium	Medium and small	Medium and small	Big, medium and small	Medium and small	Big and medium
Morphology	Black mantle	White mantle and spiral horns	White mantle and long ears	White mantle with black head and neck	Red mantle	White mantle
Female BW, kg	35	46	48	46	48	60
Fertility rate, %${}^{a}$	95	95	97	95	95	90
Prolificacy${}^{b}$	1.6	1.8	2.2	1.8	2.1	1.6
Age at first kidding, months	18	15	15	18	15	12
Days in milk	210${}^{c}$	210${}^{d}$	210${}^{d}$	210${}^{d}$	210${}^{d}$	280${}^{e}$
Milk yield, kg lactation${}^{-1^{c}}$	Parity 1=117 Parity 2=126 Parity ≥3=162	Parity 1=282 Parity 2=327 Parity ≥3=359	Parity 1=158 Parity 2=407 Parity ≥3=336	Parity 1=242 Parity 2=307 Parity ≥3=358	Parity 1=121 Parity 2=337 Parity ≥3=452	Parity 1=373 Parity 2=569 Parity ≥3=613

BW: body weight. ${}^{a}$ Fertility rate: number of kidding does/number of inseminated does. ${}^{b}$ Prolificacy: number kids/kidding. ${}^{c}$ Information retrieved from Noè et al. (2005). ${}^{d}$ Information retrieved from Di Trana et al. (2015). ${}^{e}$ Information retrieved from Amicabile (2016).

### 2.2 Sample collection and analysis

The milk yield (kg day${}^{-1}$) of individual does, as the sum of morning and evening milkings, was recorded every 2–3 weeks from 2 to 30 weeks of lactation using the recording jars in the milking parlour and an individual milk sample (50 mL; n=840) collected during the morning milking. Thereafter, milk samples were stored in portable refrigerators (4 ${}^{\circ }$C) and transferred to the milk laboratory of the Breeders Association of the Basilicata region (Potenza, Italy). Samples were warmed at 37 ${}^{\circ }$C in a water bath prior to milk analysis for fat, protein and lactose percentages with MilkoScan FT6000 (Foss Electric, Hillerød, Denmark). Fat-corrected milk at 3.5 % (FCM, kg day${}^{-1}$) was calculated according to Pulina et al. (1991) following Eq. (1):

1 FCM=milk yield×(0.634+0.1046×fat).

Somatic cell count (SCC, cells mL${}^{-1}$) was determined using Fossomatic FC (Foss Electric, Hillerød, Denmark) and transformed to SCS according to Wiggans and Shook (1987) using the following Eq. (2):

2 $$\mathrm{SCS}=3+\mathrm{log}2\;\left(\mathrm{SCC}/100\;000\right).$$

### 2.3 Statistical analysis

Data were analyzed using the MIXED procedure of SAS v. 9.4 (SAS Institute Inc., Cary, NC), with repeated measures. The statistical analyses included breed, week of lactation, and the interaction between breed and week of lactation as fixed effects and animal effect nested within breed and residual as random effects. Residual distributions from the model for each trait were examined and outliers were removed. The final mixed model was performed on 815 records. When the F ratio was significant, multiple comparisons of least squares means (LSMs) were performed using Bonferroni's test adjustment. Values are shown as LSM ± SE and significance was declared at p<0.05 unless otherwise indicated.

## 3 Results and discussion

The analysis of variance revealed that milk yield, composition and SCS were affected (p<0.001) by breed and week of lactation. The interaction between breed and week of lactation was significant for all the studied traits with the exception of SCS.

**Figure 1:**
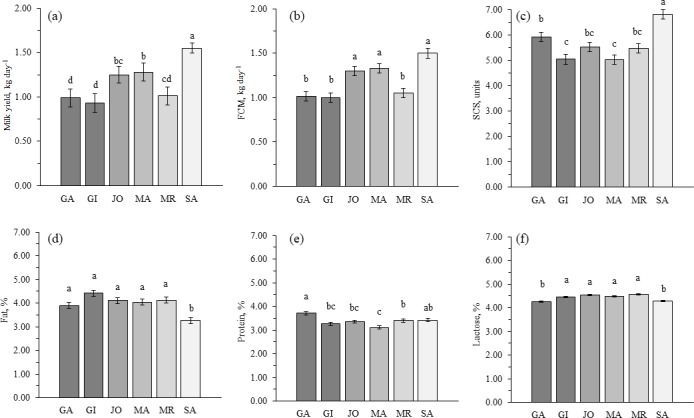
Least squares means (with SE) for **(a)** milk yield, **(b)** fat-corrected milk at 3.5 % (FCM), **(c)** somatic cell score (SCS), **(d)** fat percentage, **(e)** protein percentage, and **(f)** lactose percentage for Garganica (GA), Girgentana (GI), Jonica (JO), Maltese (MA), Mediterranean Red (MR) and Saanen (SA) goat breeds. Means with different letters within a trait are significantly different (p<0.05).

Least squares means of the studied traits for breed effect are shown in Fig. 1. Saanen yielded between 0.27 and 0.62 kg day${}^{-1}$ more milk than the local breeds (p<0.05). Among local breeds, MA and JO were the most productive, with an average of 1.28±0.05 and 1.25±0.05 kg day${}^{-1}$, respectively, while MR, GA and GI produced ≤1.00 kg day${}^{-1}$. Saanen is one of the most specialized dairy breeds worldwide, and it has been subjected to intensive genetic improvement for milk yield resulting in more days in milk and greater milk yield than other breeds (Serradilla, 2001). Serradilla (2001) has reported that usually local breeds exhibit lower lactation milk yield than cosmopolitan dairy specialized breeds. However, after standardizing milk production with a 3.5 % title of fat, MA and JO production was similar to that of SA (Fig. 1).

The greatest SCS was observed for SA (6.81±0.18; p<0.05) and among local breeds GA (5.92±0.18) showed higher SCS than GI and MA (5.03±0.18; p<0.05; Fig. 1). Higher values of SCC in goat milk compared to cow milk is physiological because goat milk protein secretion follows an apocrine process resulting in the release of the apical part of epithelial cells (Jiménez-Granado et al., 2014), while cows follow a merocrine process. In small ruminants, Raynal-Ljutovac et al. (2007) and Jiménez-Granado et al. (2014) reported that non-pathological factors such as breed, parity, stage of lactation, type of birth, oestrus, and diurnal, monthly and seasonal variations are responsible for 48 % of SCC variance in dairy sheep and goats. However, studies on goat milk seem to confirm a negative relationship between high SCC and milk yield and quality which could affect milk rennet ability and cheese yield (Raynal-Ljutovac et al., 2007).

Fat, protein and lactose content (Fig. 1) were in agreement with those reported by FAO (2013) for goat milk. Milk fat content was similar among local breeds, whereas SA produced approximately 20 % less milk fat than the average of the local breeds (p<0.05; Fig. 1). The lower milk fat content of SA goats compared to the Italian local breeds is in agreement with Landau et al. (1995) and Donkin et al. (1996), who compared SA with Israeli and South African local goat breeds, respectively. As reviewed by Goetsch et al. (2011), the lower fat content in milk of SA (3.26±0.12 %; Fig. 1) compared with local breeds could be partially explained by a dilution effect, where SA yielded on average 30 % more milk per day than the local breeds. Additionally, Prasad et al. (2005) reported that the higher the milk production level, the lower the concentration of fat. Among local breeds, milk of GA had the greatest protein content (3.71±0.07 %; p<0.05). Moreover, SA showed a protein content (3.42±0.07 %) similar to the local breeds, with the exception of MA (3.11±0.07 %; p<0.05), which was lower (Fig. 1). These results disagreed with Tripaldi et al. (1998) and Serradilla (2001), who reported a lower protein content for SA compared to other local breeds. Moreover, Tripaldi et al. (1998) reported no differences in the protein content in milk of GA, MA and MR breeds. Lactose content was similar among GI, IO, MA and MR (4.51±0.04 %), while GA and SA (4.26±0.03 %) presented a lower content (p<0.05; Fig. 1). The goat breeds which showed the greatest lactose content were the ones with the lowest SCS, in agreement with Sung et al. (1999), who reported a negative correlation between SCS and lactose content among cosmopolitan breeds in Taiwan. The decrease in lactose percentage is usually related to mastitis occurrence due to the decrease in the synthesis function of the mammary gland (Raynal-Ljutovac et al., 2007). Nevertheless, although some authors have reported a decrease in lactose concentration with an increase in SCC in goat milk (Zeng et al., 1997; Sung et al., 1999), others have not observed any effects (Raynal-Ljutovac et al., 2007).

**Figure 2:**
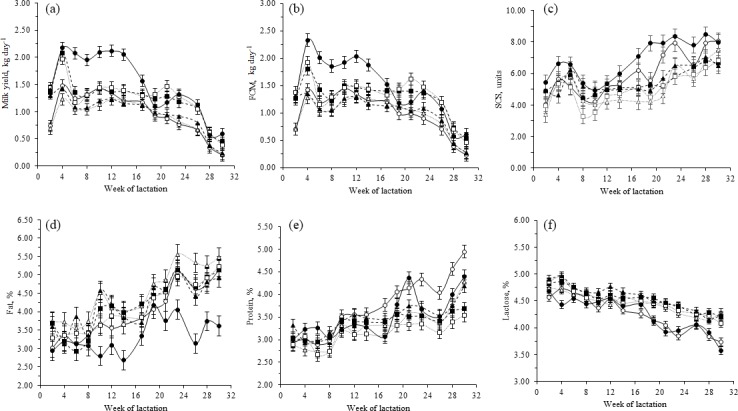
Least squares means (with SE) for **(a)** milk yield, **(b)** fat-corrected milk at 3.5 % (FCM), **(c)** somatic cell score (SCS), **(d)** fat percentage, **(e)** protein percentage, and **(f)** lactose percentage for Garganica, Girgentana, Maltese, Saanen, Mediterranean Red and Jonica goat breeds across lactation. Garganica (○), Girgentana (△), Maltese (□), Saanen (•), Mediterranean Red (▴), and Jonica (▪) goat breeds across lactation.

The variation in milk yield and quality traits across weeks of lactation followed the general pattern described for small ruminants. In particular, milk yield increased in early lactation until the peak at approximately 4 weeks after kidding, followed by a decline towards the end of lactation (after 24 weeks) as shown in Fig. 2. The pattern of milk yield during the lactation was quite similar among breeds, with SA producing more milk between week 6 and 14 compared with local breeds (p<0.05). Differences after the peak of lactation between the SA and the local breeds were less evident when comparing FCM (Fig. 2). Gipson and Grossman (1989) reported slightly earlier peak milk yield in Alpine (43.4 days) and Toggenburg (50.7 days) goat breeds compared with our findings (28 days). Girgentana and MR breeds after peak milk yield maintained a persistent lactation, keeping their production stable until 23 weeks of lactation, followed by SA and GA breeds (16 and 20 weeks, respectively). The breeds that were less persistent after peak milk yield were JO and MA.

The increase in SCS, fat and protein towards the end of the lactation, when milk volume is low (Fig. 2), has been explain by several authors in part as a concentration effect (Goetsch et al., 2011; Mestawet et al., 2012; Jiménez-Granado et al., 2014). Although SA showed greater SCS than local breeds throughout lactation, these differences were rarely significant (Fig. 2). Fat content of SA was significantly lower during mid and late lactation compared to the local breeds (Fig. 2). Protein content showed significant differences only in late lactation with the greatest content for GA breed (Fig. 2). Lactose content decreased gradually until the end of lactation (Fig. 2), being this decrease in late lactation (from the 18th to 30th week) less marked for GI, JO, MA and MR than for GA and SA goat breeds. The decrease in lactose content at the end of lactation has been also reported by Prasad et al. (2005) in Indian goat breeds. However, Zeng et al. (1997) have reported a more constant lactose content through the lactation of Alpine goats. Overall, differences between SA and local breeds for milk yield, SCS and milk composition were consistent across lactation.

## 4 Conclusions

Our results contributed to the characterization of milk yield and composition of local Italian goat breeds. Results indicated that breed and week of lactation were the main factors responsible for the variation in the studied traits. Local breeds produced less milk but with lower SCS, greater fat and lactose content than the SA cosmopolitan breed. Overall, no differences in protein percentage were observed between SA and local breeds, which is an important trait for cheese transformation. Therefore, the variability of milk yield and composition traits reported in the present study are of interest to preserve the biodiversity of local goat breeds and for the dairy industry to balance milk volume and component concentration for cheese manufacturing. Further studies on the milk fat profile and mineral composition of local goat breeds would be beneficial due to their relationships with human health and their milk rennetability.

## Data Availability

The data of the paper are available upon request from the corresponding author.
